# Two transgenic mouse models for β-subunit components of succinate-CoA ligase yielding pleiotropic metabolic alterations

**DOI:** 10.1042/BCJ20160594

**Published:** 2016-10-11

**Authors:** Gergely Kacso, Dora Ravasz, Judit Doczi, Beáta Németh, Ory Madgar, Ann Saada, Polina Ilin, Chaya Miller, Elsebet Ostergaard, Iordan Iordanov, Daniel Adams, Zsuzsanna Vargedo, Masatake Araki, Kimi Araki, Mai Nakahara, Haruka Ito, Aniko Gál, Mária J. Molnár, Zsolt Nagy, Attila Patocs, Vera Adam-Vizi, Christos Chinopoulos

**Affiliations:** 1 Department of Medical Biochemistry, Semmelweis University, Tuzolto Street 37-47, Budapest 1094, Hungary; 2 MTA-SE Lendület Neurobiochemistry Research Group, Budapest 1094, Hungary; 3 Monique and Jacques Roboh Department of Genetic Research and the Department of Genetic and Metabolic Diseases, Hadassah-Hebrew University Medical Center, Jerusalem 91120, Israel; 4 Department of Clinical Genetics, Copenhagen University Hospital Rigshospitalet, Copenhagen 2100, Denmark; 5 MTA-SE Lendület Ion Channel Research Group, Budapest 1094, Hungary; 6 Institute of Resource Development and Analysis, Kumamoto University, 2-2-1 Honjo, Chuo-ku, Kumamoto 860-0811, Japan; 7 Institute of Genomic Medicine and Rare Disorders, Semmelweis University, Budapest 1083, Hungary; 8 MTA-SE Lendület Hereditary Endocrine Tumours Research Group, Budapest 1088, Hungary; 9 MTA-SE Laboratory for Neurobiochemistry, Budapest 1094, Hungary

**Keywords:** inborn error of metabolism, mitochondrial dysfunction, mouse genetics, mtDNA

## Abstract

Succinate-CoA ligase (SUCL) is a heterodimer enzyme composed of Suclg1 α-subunit and a substrate-specific Sucla2 or Suclg2 β-subunit yielding ATP or GTP, respectively. In humans, the deficiency of this enzyme leads to encephalomyopathy with or without methylmalonyl aciduria, in addition to resulting in mitochondrial DNA depletion. We generated mice lacking either one *Sucla2* or *Suclg2* allele. *Sucla2* heterozygote mice exhibited tissue- and age-dependent decreases in Sucla2 expression associated with decreases in ATP-forming activity, but rebound increases in cardiac Suclg2 expression and GTP-forming activity. Bioenergetic parameters including substrate-level phosphorylation (SLP) were not different between wild-type and *Sucla2* heterozygote mice unless a submaximal pharmacological inhibition of SUCL was concomitantly present. mtDNA contents were moderately decreased, but blood carnitine esters were significantly elevated. *Suclg2* heterozygote mice exhibited decreases in Suclg2 expression but no rebound increases in Sucla2 expression or changes in bioenergetic parameters. Surprisingly, deletion of one *Suclg2* allele in *Sucla2* heterozygote mice still led to a rebound but protracted increase in Suclg2 expression, yielding double heterozygote mice with no alterations in GTP-forming activity or SLP, but more pronounced changes in mtDNA content and blood carnitine esters, and an increase in succinate dehydrogenase activity. We conclude that a partial reduction in Sucla2 elicits rebound increases in Suclg2 expression, which is sufficiently dominant to overcome even a concomitant deletion of one Suclg2 allele, pleiotropically affecting metabolic pathways associated with SUCL. These results as well as the availability of the transgenic mouse colonies will be of value in understanding SUCL deficiency.

## Introduction

Succinate-CoA ligase (SUCL), also known as succinyl coenzyme A synthetase, or succinate thiokinase is a heterodimer enzyme composed of an invariant α-subunit encoded by *SUCLG1* and a substrate-specific β-subunit encoded by either *SUCLA2* or *SUCLG2*. This dimer combination results in either an ATP-forming (EC 6.2.1.5) or a GTP-forming SUCL (EC 6.2.1.4). *ΔG* of either reaction is ∼0.07 kJ/mol and therefore, reversible [42]. SUCL is located in the mitochondrial matrix catalyzing the conversion of succinyl-CoA and ADP (or GDP) to CoASH, succinate and ATP (or GTP) [30]. As such, it is at the intersection of several metabolic pathways [71]: (i) it is part of the citric acid cycle, a major metabolic hub for the interconversion of many metabolites; (ii) when SUCL proceeds in the direction towards succinyl-CoA, this product may follow heme metabolism [39]; (iii) in extrahepatic tissues, succinyl-CoA will also participate in the metabolism of ketone bodies [21]; (iv) the reaction proceeding towards ATP formation termed ‘substrate-level phosphorylation’ (SLP) can yield high-energy phosphates in the absence of oxygen [11,13,35], whereas GTP-forming SUCL may support ATP formation through concerted action with a mitochondrial nucleotide diphosphate kinase which complexates with either ATP- or GTP-forming SUCL [31,32,38]; (v) exactly because of the association of SUCL with the nucleotide diphosphate kinase, SUCL is important in maintaining mtDNA content through provision of phosphorylated deoxyribonucleotides [69]; (vi) succinyl-CoA is the entry point to the citric acid cycle in the catabolism of certain biomolecules (methionine, threonine, isoleucine, valine, propionate, odd chain fatty acids and cholesterol) through propionyl → methylmalonyl → to succinyl-CoA mediated by the sequential actions of propionyl-CoA carboxylase and methylmalonyl-CoA mutase [53]; (vii) in turn, increases in propionyl-CoA and methylmalonyl-CoA may cause secondary metabolic aberrations due to their ability to inhibit steps in urea cycle, gluconeogenesis and the glycine cleavage system [65]; (viii) in specialized cells of the brain, succinate is the entry point to the citric acid cycle of the ‘GABA shunt’ from succinate semialdehyde, a metabolite which is also in equilibrium with γ-hydroxybutyric acid [28,55,59] and (ix) in cells of macrophage lineage, SUCL metabolizes endogenously produced itaconate to itaconyl-CoA [51]. Furthermore, succinyl-CoA has been recently reported to serve as a cofactor for lysine succinylation, a wide-spread posttranslational modification [77], and succinate to be a metabolic signal in inflammation [48,70]. Finally, succinate has been branded as an ‘oncometabolite’ linking the citric acid cycle to hypoxia and oncogenesis [11,66,67].

In view of the involvement of SUCL in all of the above, it is not surprising that its deficiency leads to pleiotropic pathology, which is also influenced by the tissue-specific expression of its subunits: SUCLA2 is strongly expressed in skeletal muscle, brain and heart, whereas SUCLG2 is barely detected in brain and muscle, but robustly expressed in liver and kidney [40]. Furthermore, in the human brain, SUCLA2 is exclusively expressed in the neurons, whereas SUCLG2 is only found in cells forming the microvasculature [16,17].

To date, 51 patients have been reported with SUCLA2 deficiency [6,7,20,24,29,41,44,46,49,50,52,54], and 21 patients with SUCLG1 deficiency, due to different mutations [7]. Patients with *SUCLG1* mutations may have an extremely severe phenotype with antenatal manifestations of the disorder, severe acidosis with lactic aciduria in the first day of life and death within 2–4 days [53] or a phenotype similar to those of patients with *SUCLA2* mutations. Mutations in the *SUCLG2* gene have not been reported so far and may be incompatible with life.

SUCLA2 deficiency (MIM ID#612073) has an incidence of 1 in 1700 in the Faroe Islands due to a founder effect and a carrier frequency of 1 in 33 [54]. More recently, evidence of two founder mutations in the Scandinavian population has also been put forward [7]. The symptoms comprise hypotonia, muscle atrophy, hyperkinesia, severe hearing impairment and postnatal growth retardation. Neuroimaging findings comprise demyelination, central and cortical atrophy including atrophy of the basal ganglia [6,54]. Some of the patients fulfil the criteria for Leigh syndrome [53]. Urine and plasma methylmalonic acid, C3-carnitine and C4-dicarboxylic carnitine (the latter likely to be a mixture of succinyl and methylmalonyl carnitine ester) are elevated [53,54], while there are no abnormalities related to liver functions. Median survival is 20 years [7]; the longest documented survival is 45 years [49]. It is being hypothesized that patients with missense mutations in *SUCLA2* (or *SUCLG1*) may exhibit some residual SUCL activity that is associated with longer survivals; however, given the small number of patients and the lack of expression studies providing direct experimental evidence of residual activity, such postulations must be interpreted cautiously [7]. Heterozygous relatives of patients with SUCLA2 deficiency are asymptomatic [53,54].

Given the role of SUCL in maintaining mtDNA content, SUCLA2 deficiency patients suffer from mtDNA depletion in muscle [50,53,54]. Data from heart and brain biopsies are not available. mtDNA depletion has also been reported in fibroblasts but only from some patients [6], or only after serum deprivation [47]. mtDNA depletion would influence many targets in mitochondria, including the electron transport complexes of the respiratory chain creating a bioenergetic insufficiency that could in turn impair energy-dependent mechanisms. Indeed, respiratory chain enzyme analysis show decreased complex I, III and IV activity, whereas complex II, which is encoded exclusively by nDNA genes, shows normal activity [7,54]. There are no data available regarding ETC activities from brain tissue. In fibroblasts, a slightly decreased complex IV activity has been reported [54].

Here, we generated transgenic mice lacking either one *Sucla2* or one *Suclg2* allele. Homozygous knockout mice for either gene were never born. We quantitated the expression of SUCL subunits in mitochondria isolated from brains, hearts and livers of 3-, 6- and 12-month-old wild-type (WT) and heterozygote mice. In the tissues of Sucla2 heterozygote mice, we investigated respiration rates and membrane potential (ΔΨm) for an array of mitochondrial substrates and various metabolic states, complex I, II, II/III and IV activities, as well as SLP during respiratory inhibition or true anoxia. SLP was further investigated during submaximal inhibition of SUCL by either itaconate or KM4549SC. We also compared mtDNA content, ATP-forming and GTP-forming SUCL activities, and blood levels of 20 carnitine esters. Furthermore, we cross-bred Sucla2+/− with Suclg2+/− mice, yielding double heterozygote Sucla2+/−/Suclg2+/− mice, and investigated the expression of g1, g2 and a2 subunits, mtDNA content, blood carnitine esters and bioenergetic parameters.

Our results show that, in Sucla2 heterozygote mice, there is a rebound increase in Suclg2 expression associated with mostly unaffected bioenergetic parameters, while mtDNA contents are moderately decreased in some organs, and blood carnitine levels are elevated. The rebound increase in Suclg2 expression due to deletion of one *Sucla2* allele was so dominant that it was even observed — albeit to a moderate extent — in double heterozygote Sucla2+/−/Suclg2+/− mice. Results obtained from embryonic tissues of Sucla2−/− mice have been published in ref. [18] and are discussed in relation to the results obtained here.

## Experimental procedures

### Animals

Mice were of either 129/SvEv (Sucla2 heterozygote strain) or C57Bl/6N (Suclg2 heterozygote strain) background. The animals used in our study were of either sex and of 3, 6 or 12 months of age. Mice were housed in a room maintained at 20–22°C on a 12-h light–dark cycle with food and water available *ad libitum*. All experiments were approved by the Animal Care and Use Committee of the Semmelweis University (Egyetemi Állatkísérleti Bizottság) and the EU Directive 2010/63/EU for animal experiments. Sucla2 heterozygous mice were generated by Texas A&M Institute for Genomic Medicine (TIGM). Suclg2 heterozygote mice [B6-Suclg2^Gt(pU-21KBW)131Card^] were generated at CARD, Kumamoto University, Japan. For further details regarding the generation of these two mouse colonies, see the Results section. Neither Sucla2 −/− nor Suclg2 −/− mice were ever born from mating heterozygous mice, suggesting that the complete absence of either gene is incompatible with life in mice, and as also reported in ref. [18]. By mating Sucla2 heterozygous mice with Suclg2 heterozygous mice, double transgenic (Sucla2+/−/Suclg2+/−) mice were born and viable.

### Isolation of mitochondria

Isolation of mitochondria from mouse liver, heart and brain: liver and heart mitochondria from all animals were isolated as described in ref. [72], with the modifications described in refs [13] and [15]. Nonsynaptic brain mitochondria were isolated on a Percoll gradient as described previously [68], with minor modifications detailed in ref. [14]. Protein concentration was determined using the bicinchoninic acid assay and calibrated using bovine serum standards using a Tecan Infinite® 200 PRO series plate reader (Tecan Deutschland GmbH, Crailsheim, Germany). Yields were typically 0.2 ml of ∼20 mg/ml per two brains; for liver, yields were typically 0.7 ml of ∼70 mg/ml per two livers, and for heart mitochondria, yields were typically 0.1 ml of ∼15 mg/ml per two hearts.

### Determination of membrane potential in isolated liver mitochondria

ΔΨm of isolated mitochondria [0.5–1 mg — depending on the tissue of origin — per two ml of medium containing (in mM): KCl 8, K-gluconate 110, NaCl 10, HEPES 10, KH_2_PO_4_ 10, EGTA 0.005, mannitol 10, MgCl_2_ 1, substrates as indicated in the figure legends, 0.5 mg/ml bovine serum albumin (fatty acid-free), pH 7.25 and 5 µM safranin O] was estimated fluorimetrically with safranin O [1]. Traces obtained from mitochondria were calibrated to millivolts as described in ref. [13]. Fluorescence was recorded in a Hitachi F-7000 spectrofluorimeter (Hitachi High Technologies, Maidenhead, UK) at a 5-Hz acquisition rate using 495- and 585-nm excitation and emission wavelengths, respectively, or at a 1-Hz rate using the O2k-Fluorescence LED2-Module of the Oxygraph-2k (Oroboros Instruments, Innsbruck, Austria) equipped with an LED exhibiting a wavelength maximum of 465 ± 25 nm (current for light intensity adjusted to 2 mA, i.e. level ‘4’) and an <505 nm shortpass excitation filter (dye-based, filter set ‘Safranin’). Emitted light was detected by a photodiode (range of sensitivity: 350–700 nm), through an >560 nm longpass emission filter (dye-based). Experiments were performed at 37°C. Safranin O is known to exert adverse effects on mitochondria if used at sufficiently high concentrations (i.e. above 5 μM, discussed in ref. [36]). However, for optimal conversion of the fluorescence signal to ΔΨm, a concentration of 5 μM safranin O is required, even if it leads to diminution of the respiratory control ratio by approximately one unit (not shown). Furthermore, the nonspecific binding component of safranin O to mitochondria (dictated by the mitochondria/safranin O ratio) was within 10% of the total safranin O fluorescence signal, estimated by the increase in fluorescence caused by the addition of a detergent to completely depolarized mitochondria (not shown). As such, it was accounted for, during the calibration of the fluorescence signal to ΔΨm.

### Mitochondrial respiration

Oxygen consumption was estimated polarographically using an Oxygraph-2k. Depending on the tissue of origin, mitochondria (0.5–1 mg) were suspended in 2 ml of incubation medium, the composition of which was identical with that for ΔΨm determination. Experiments were performed at 37°C. Oxygen concentration and oxygen flux (pmol s^−1^ mg^−1^; negative time derivative of oxygen concentration, divided by mitochondrial mass per volume and corrected for instrumental background oxygen flux arising from oxygen consumption of the oxygen sensor and back-diffusion into the chamber) were recorded using DatLab software (Oroboros Instruments).

### Cell cultures

Fibroblast cultures from skin biopsies from the patient with no SUCLA2 expression and a control subject were prepared. Cells were grown on poly-l-ornithine-coated flasks for 5–7 days in RPMI1640 medium (GIBCO, Life Technologies, Carlsbad, CA, USA) supplemented with 10% fetal bovine serum and 2 mM glutamine and kept at 37°C in 5% CO_2_. The medium was also supplemented with penicillin, streptomycin and amphotericin (Sigma-Aldrich, St. Louis, MO, USA).

### Mitochondrial membrane potential determination in *in situ* mitochondria of permeabilized fibroblast cells

Mitochondrial membrane potential (ΔΨm) was estimated using fluorescence quenching of the cationic dye safranin O due to its accumulation inside energized mitochondria [1]. Fibroblasts were harvested by trypsinization, permeabilized as detailed in ref. [33] and suspended in a medium identical with that as for ΔΨm measurements in isolated mitochondria. Substrates were 5 mM glutamate and 5 mM malate. Fluorescence was recorded in a Tecan Infinite® 200 PRO series plate reader using 495 and 585 nm excitation and emission wavelengths, respectively. Experiments were performed at 37°C.

### Western blot analysis

Isolated mitochondria were solubilized in RIPA buffer containing a cocktail of protease inhibitors (Protease Inhibitor Cocktail Set I, Merck Millipore, Billerica, MA, USA) and frozen at −80°C for further analysis. Frozen pellets were thawed on ice, and their protein concentration was determined using the bicinchoninic acid assay as detailed above, loaded at a concentration of 3.75 µg per well on the gels and separated by sodium dodecyl sulfate–polyacrylamide gel electrophoresis (SDS–PAGE). Separated proteins were transferred onto a methanol-activated polyvinylidene difluoride membrane. Immunoblotting was performed as recommended by the manufacturers of the antibodies. Rabbit polyclonals anti-SUCLG1, anti-SUCLG2, anti-voltage-dependent anion channel 1 (VDAC1; Abcam, Cambridge, UK), and anti-SUCLA2 (Proteintech Europe Ltd, Manchester, UK) primary antibodies were used at titers of 1:5000. Immunoreactivity was detected using the appropriate peroxidase-linked secondary antibody (1:5000, donkey anti-rabbit Jackson Immunochemicals Europe Ltd, Cambridgeshire, UK) and enhanced chemiluminescence detection reagent (ECL system; Amersham Biosciences GE Healthcare Europe GmbH, Vienna, Austria). Densitometric analysis of the bands was performed in Fiji [64].

### mtDNA content

Total DNA was isolated from four pooled tissues from each mouse group using the QIAamp DNA Mini Kit (QIAGEN) following the manufacturer's instructions. Relative mtDNA content was quantified in triplicate by real-time PCR using primers for cox1 (forward primer 5′-TGCTAGCCGCAGGCATTA C-3′; reverse primer 5′-GGGTGCCCAAAGAATCAGAAC-3′) and normalized against the nuclear-encoded actinB gene (forward primer 5′-GGAAAAGAGCCTCAGGGCAT-3′, reverse primer 5′-GAAGAGCTATGAGCTGCCTGA-3′), as previously described [76]. DNA was amplified in an ABI 7900 system as follows: 95°C for 10 min followed by 45 cycles of a two-stage temperature profile of 95°C for 15 s and 60°C for 1 min.

### Protein purification

The gene sequences for mature human SUCLG1 (residues 29–333, ∼33.2 kDa, GenBank: CAG33420.1) and mature human SUCLG2 (residues 39–432, ∼43.6 kDa, GenBank: AAH68602.1) were sequence-optimized for expression in *Escherichia coli*, synthesized, incorporated in pJ411 plasmids bearing kanamycin resistance, and sequence-verified (DNA2.0, Newark, CA, USA). The native protein sequence in each case was supplemented with a C-terminal hexahistidine tag (GSHHHHHH). Each pJ411-SUCLG1/2 plasmid was transfected into inducible *E. coli* BL21 (DE3) strain and the bacteria were grown in Luria-Bertani medium at 37°C. Protein expression was induced with 1 mM isopropyl β-d-1-thiogalactopyranoside for 3 h. The collected bacteria were sonicated in 10 ml of lysis buffer [25 mM Tris (pH 8.5), 150 mM NaCl, 0.5 mg/ml lysozyme, 0.2% Triton X-100] per gram of wet pellet. Both proteins formed inclusion bodies when overexpressed, with minimal or no presence in the soluble fraction of the lysate. The proteins were purified in their unfolded state (7 M urea and 200 mM NaCl) with affinity chromatography, after binding to Ni-Sepharose™ 6 Fast Flow resin (GE Healthcare). The eluates were diluted 15-fold in 20 mM Tris (pH 8.5) and 100 mM NaCl, the precipitated protein was removed and the supernatants were dialyzed against the same buffer. The purity of the two proteins was assessed with SDS–PAGE and the final protein concentrations were estimated using the bicinchoninic acid assay as detailed above. The protein stocks were aliquoted, flash-frozen in liquid nitrogen and stored at −80°C.

### Electron transport chain complex and citrate synthase activity assays

Enzymatic activities of rotenone-sensitive NADH CoQ reductase (complex I), succinate cytochrome *c* reductase (complex II/III), succinate dehydrogenase (complex II), cytochrome *c* oxidase (complex IV) and citrate synthase (CS), a mitochondrial marker enzyme, were determined in isolated mitochondria as we have previously described [58,60].

### Determination of SUCL activity

ATP- and GTP-forming SUCL activity in isolated mitochondria was determined at 30°C, as described in ref. [3], with the modifications detailed in ref. [40]. Mitochondria (0.25 mg) were added in an assay mixture (2 ml) containing 20 mM potassium phosphate, pH 7.2, 10 mM MgCl_2_ and 2 mM ADP or GDP. The reactions were initiated by adding 0.2 mM succinyl-CoA and 0.2 mM DTNB [5,5′-dithiobis(2-nitrobenzoic acid)] in quick succession. The molar extinction coefficient value at 412 nm for the 2-nitro-5-thiobenzoate anion formed upon reaction of DTNB with CoASH was considered as 13 600 M^−1^ cm^−1^. Rates of 2-nitro-5-thiobenzoate formation were followed spectrophotometrically during constant stirring.

### Determination of acylcarnitines

Multiple reaction monitoring transitions of butyl ester derivatives of acylcarnitines from dry bloodspots and stable isotope internal standards were analyzed by electrospray ionization tandem mass spectrometry (MS–MS) using a Waters Alliance 2795 separations module coupled to a Waters Micromass quarto micro API mass spectrometer monitoring for acylcarnitines (Milford, MA, USA), as described in ref. [57].

### Determination of Sucla2 mRNA by quantitative real-time PCR

mRNA coding for Sucla2 was quantified by qPCR in two different laboratories using two different ‘housekeeping’ mRNAs for normalization, β-actin or proteasome 26S subunit, ATPase 4 (Psmc4). In both cases, total RNA was isolated from the organs (livers, hearts and brains) of at least four mice per age group and genotype (WT or Sucla2+/−) with the RNeasy Micro Kit (Qiagen, Hilden, Germany) according to the manufacturer's instructions. RNA (1 µg) was reverse-transcribed with the QuantiTect Reverse Transcription Kit (Qiagen). Subsequently, quantitative real-time PCR was carried out using predesigned TaqMan Gene Expression Assays (Thermo Fisher Scientific, Waltham, MA, USA): Sucla2 (Mm01310541_m1) and Actb (Mm00607939_s1). Real-time reaction was performed on a QuantStudio 7 Flex Real-Time PCR system (Applied Biosystems, Life Technologies, Carlsbad, CA, USA) according to the manufacturer's protocol. Gene expression level was normalized to β-actin. Fold change was calculated using the 2-ΔΔ*C*_t_ method [8]. Alternatively, expression level of Sucla2 mRNA was determined by real-time PCR using the TaqMan Gene Expression assay kit and 7500 Real-Time PCR System (Applied Biosystems), using the TaqMan Gene Expression Assays, XS, Sucla2 (AB, 4331182, FAM/MGB-NFQ) kit. Measured values were normalized by using the TaqMan Gene Expression Controls, Psmc4 mouse (AB, 4448489, VIC-MGB) kit, as recommended by Applied Biosystems for standard gene expression experiments because of their design criteria.

### Statistics

Data are presented as averages ± SEM or SD where indicated. Significant differences between two groups were evaluated by Student's *t*-test; significant differences between three or more groups were evaluated by one-way analysis of variance followed by Tukey's or Dunnett's *post hoc* analysis. A value of *P* < 0.05 was considered statistically significant. *P < 0.05, **P < 0.01 and ***P < 0.001. If normality test failed, ANOVA on ranks was performed. Wherever single graphs are presented, they are representative of at least three independent experiments.

### Reagents

Standard laboratory chemicals, enzyme substrates and itaconic acid were from Sigma-Aldrich. SF 6847 was from Enzo Life Sciences (ELS AG, Lausen, Switzerland). Carboxyatractyloside (cATR) was from Merck (Merck KGaA, Darmstadt, Germany). KM4549SC (LY266500) was from Molport (SIA Molport, Riga, Latvia). Mitochondrial substrate stock solutions were dissolved in bi-distilled water and titrated to pH 7.0 with KOH. ADP was purchased as a K^+^ salt of the highest purity available (Merck) and titrated to pH 6.9. TaqMan Gene Expression Assays, XS, Suclg2 (AB, 4448892, FAM/MGB-NFQ) kit and Actb (AB, 4448489, VIC-MGB) kit were from Thermo Fisher Scientific. qPCR mix was qPCRBIO SyGreen Mix Hi-Rox (PCR Biosystems).

## Results

### Generation of Sucla2 mutant mice, Sucla2 mRNA quantification, SUCL subunit expression and enzymatic activities in WT vs. Sucla2+/− mice

**Figure 1.**
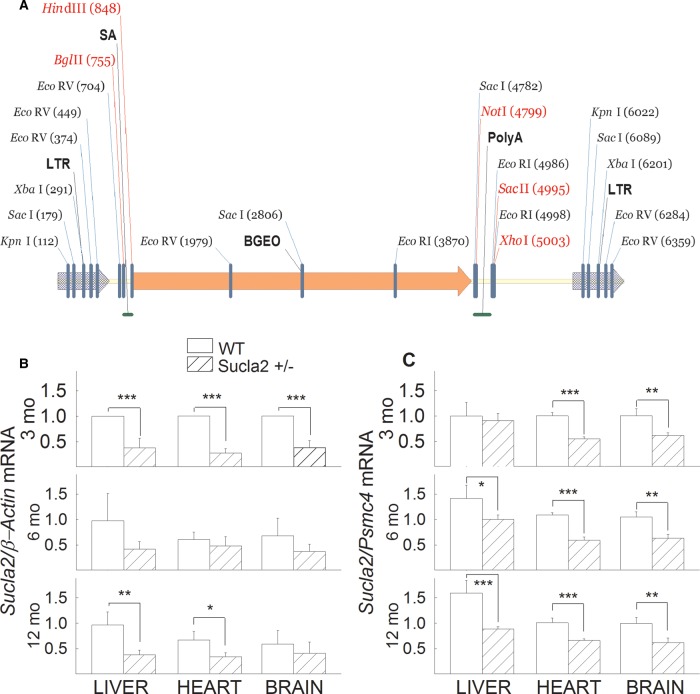
Generation of Sucla2 mutant mice and *Sucla2* mRNA quantification. (**A**) Gene trap vector for generating Sucla2 mutant mice. (**B**) Bar graphs of qPCR of *Sucla2* mRNA ratioed to *β-actin* mRNA of 3-, 6- and 12-month-old WT and Sucla2+/− mice from liver, heart and brain. (**C**) Bar graphs of qPCR of *Sucla2* mRNA ratioed to *Psmc4* mRNA of 3-, 6- and 12-month-old WT and Sucla2+/− mice from liver, heart and brain. *P < 0.05, **P < 0.01 and ***P < 0.001. Data are SEM from four different organs per animal group.

Mutant Sucla2 mice were generated using a gene-trapping technique [26]. Mice (strain C57BL/6N) were cloned from an ES cell line (IST10208H1; TIGM). The ES cell clone contained a retroviral insertion in the Sucla2 gene (intron 4) identified from the TIGM gene trap database and was microinjected into C57BL/6 albino host blastocysts to generate germline chimaeras using standard procedures. The retroviral OmniBank Vector 76 contained a splice acceptor (Figure 1A) followed by a selectable neomycin resistance marker/LacZ reporter fusion (β-Geo) for identification of successful gene trap events further followed by a polyadenylation signal. Insertion of the retroviral vector into the Sucla2 gene led to the splicing of the endogenous upstream exons into this cassette to produce a fusion that leads to termination of further transcription of the endogenous Sucla2 exons downstream of the insertion. Chimaeric males were bred to 129/SvEv females for germline transmission of the mutant Sucla2 allele.

**Figure 2.**
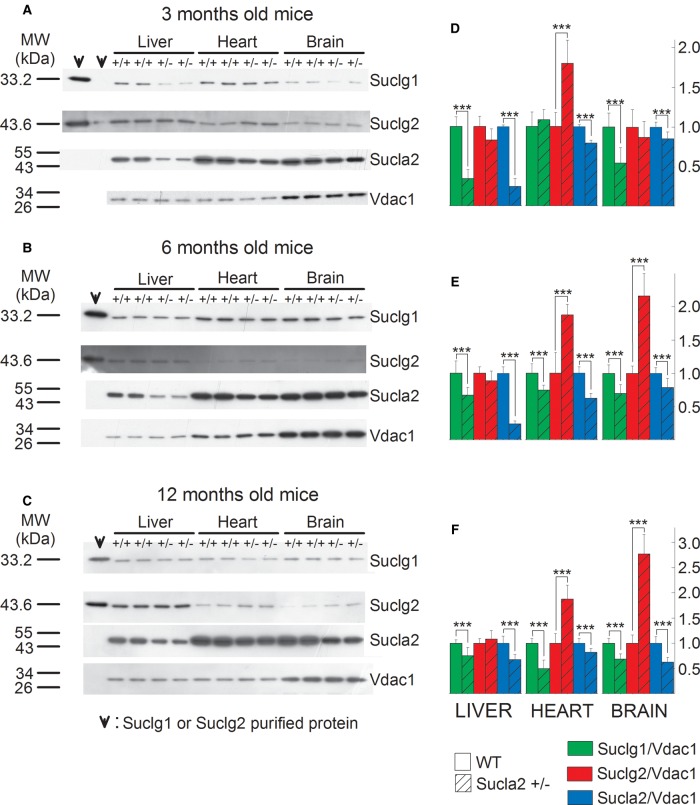
SUCL subunit expression in WT vs. Sucla2+/− mice. (**A**–**C**) Scanned images of Western blotting of purified SUCLG1 and SUCLG2 and mitochondria of 3-, 6- and 12-month-old WT and Sucla2+/− mice from liver, heart and brain. (**D**–**F**) Band density quantification of the scanned images shown in **A**–**C**, respectively. Data were arbitrarily normalized to the average density of the first two bands of WT mice per organ. ***P < 0.001. Each Western blot lane contains mitochondria (except those containing the purified SUCLG1 or SUCLG2 proteins) pooled from two or four organs per animal group. Data shown in the bar graphs are SEM.

Total RNA was isolated from the livers, hearts and brains of 3-, 6- and 12-month-old WT and Sucla2+/− mice (four animals per group), and Sucla2 mRNA was quantified by qPCR, ratioed to β-actin (Figure 1B) or Psmc4 expression (Figure 1C). As shown in Figures 1B,C, mRNA coding for Sucla2 was significantly decreased (26–71%) in the tissues obtained from Sucla2+/− mice, compared with those obtained from WT littermates. These results are in accordance with those obtained from immunodetection of Sucla2 subunit by Western blotting. These data are depicted in Figure 2. Mitochondria were prepared from the livers, hearts and brains of 3-, 6-, and 12-month-old WT and Sucla2+/− mice and SUCLG1, SUCLA2, SUCLG2 and VDAC1 were immunodetected by Western blotting. Only 3.75 µg of purified mitochondria (pooled from mitochondria obtained from eight organs per group) were loaded on each gel lane so as not to saturate the final enhanced chemiluminescence signals (see the ‘Experimental Procedures’ section). Scanned images of representative Western blots are shown in Figure 2A–C. As shown in the first two lanes of the left topmost panel in Figure 2A, purified recombinant SUCLG1 or SUCLG2 protein has been immunodetected. Purified protein has been loaded in the leftmost lane (30 ng) and in the adjacent right one (3 ng). In the remaining subpanels of Figure 2A–C, 30 ng of either SUCLG1 or SUCLG2 was loaded. From the bands obtained from the purified proteins in relation to those obtained from the purified mitochondria, we deduce that (i) the bands detected from the mitochondrial samples corresponding to slightly lower though nearly identical molecular weight (MW) presumably due to the hexahistidine tags of the recombinant proteins genuinely represent the sought proteins and (ii) the amount of either SUCLG1 or SUCLG2 in 3.75 µg of purified mitochondria corresponds to between 3 and 30 ng. The antibody directed against SUCLA2 protein has been validated in ref. [17] using fibroblasts from a patient with *SUCLA2* deletion. Anti-VDAC1 was used as a loading control.

As shown in Figure 2A–C and from the quantification of the band densities in relation to that of VDAC1 shown in Figure 2D–F, respectively, Sucla2+/− mice exhibited up to 76% decrease in Sucla2 expression, depending on the tissue and the age of the mice. Concomitantly, Sucla2+/− mice exhibited up to 66% reduction in Suclg1 protein, but also up to 177% increase in Suclg2 protein.

**Figure 3.**
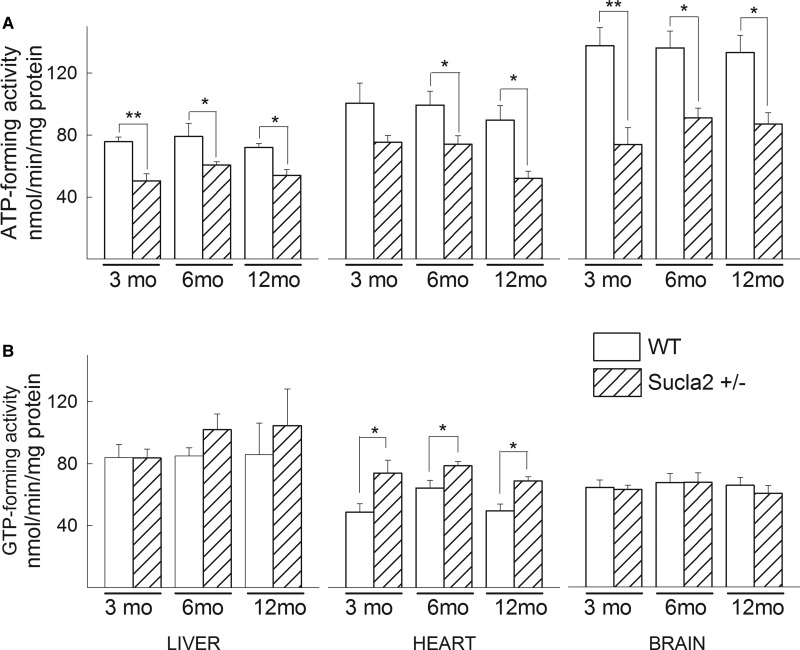
ATP- and GTP- forming SUCL activity in WT vs Sucla2+/− mice. (**A**) Bar graphs of ATP-forming SUCL activity from mitochondria of 3-, 6- and 12-month-old WT (solid) and Sucla2+/− (striped) mice from liver, heart and brain. (**B**) Bar graphs of GTP-forming SUCL activity from mitochondria of 3-, 6- and 12-month-old WT and Sucla2+/− mice from liver, heart and brain. *P < 0.05, **P < 0.01. Data shown are SEM from two or four pooled organs per animal group from four independent experiments.

In agreement with the above results regarding Suclg1/g2/a2 subunit quantification, ATP-forming activity of Sucla2+/− mice decreased, while GTP-forming activity increased, though only in heart mitochondria, for all ages (Figure 3).

From the above experiments, we obtained the information that deletion of one *Sucla2* allele is associated with a decrease in Suclg1 expression and a rebound increase in Suclg2 expression, and this is reflected in reciprocal decrease vs. increase in ATP-forming vs. GTP-forming SUCL activity.

### Generation of Suclg2 mutant mice and characterization of SUCL subunit expression and enzymatic activities of WT vs. Suclg2+/− mice

**Figure 4.**
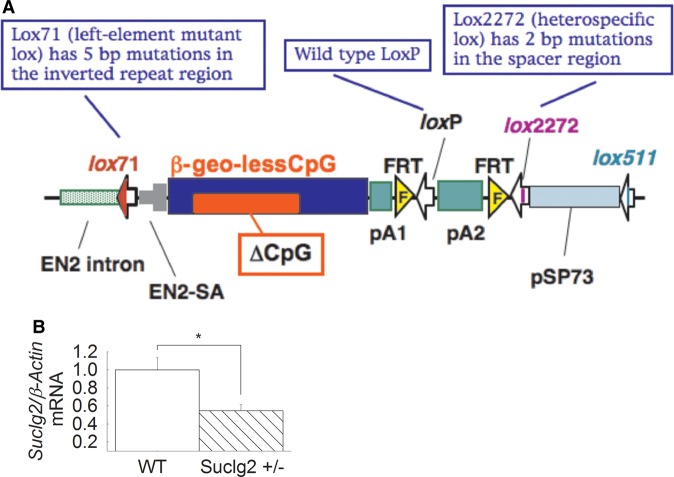
Generation of Suclg2 mutant mice and *Suclg2* mRNA quantification. (**A**) Gene trap vector for generating Suclg2 mutant mice (adapted from http://egtc.jp/action/access/vector_detail?vector=pU-21W). (**B**) *Suclg2* heterozygote ES cell line (Ayu21-KBW131: +/−) was compared with parental strain (KAB6: WT). mRNA was purified from parental ES cells (+/+) and *Suclg2* heterozygote (+/−). Suclg2 mRNA expression level was analyzed by real-time PCR using a TaqMan probe for *Suclg2* (AB, 4448892, FAM/MGB-NFQ) and normalized by a TaqMan probe for β-actin (AB, 4448489, VIC-MGB). Asterisk signifies *P*=0.039. Data shown are SEM from four independent experiments.

Mutant Suclg2 mice were generated using a gene-trapping technique [4]. Mice (strain Albino B6) were cloned from an ES cell line (Ayu21-KBW131; Exchangeable Gene Trap Clones: EGTC). The ES cell clone contained a trap vector insertion in the Suclg2 gene (first intron) identified from the EGTC database and was aggregated with morulae from ICR mice to generate germline chimaeras using standard procedures. pU21-W (accession number: AB427140, 9333 bp) was a ‘promoter trap’ vector with three stop codons, which were arranged upstream of the ATG of the β-geo in all three frames (see Figure 4A). Insertion of the trap vector into the Suclg2 gene led to the splicing of the endogenous upstream exons into this cassette to produce a fusion transcript that leads to termination of further transcription of the endogenous Suclg2 exons downstream of the insertion. Chimaeric males were bred to C57BL/6N females for germline transmission of the mutant Suclg2 allele. To investigate the expression level of Suclg2 mRNA of Suclg2 heterozygote, the original ES cell line (Ayu21-KBW131: +/−) was compared with the parental strain (KAB6: +/+). mRNA was purified from parental ES cells (+/+) and 21-KBW131 (+/−). Suclg2 expression levels of these cells were analyzed by real-time PCR using the TaqMan Gene Expression Assays, XS, Suclg2 (AB, 4448892, FAM/MGB-NFQ) kit. Heterozygous ES cells showed almost half the amount of Suclg2 mRNA compared with parent cells (Figure 4B).

**Figure 5.**
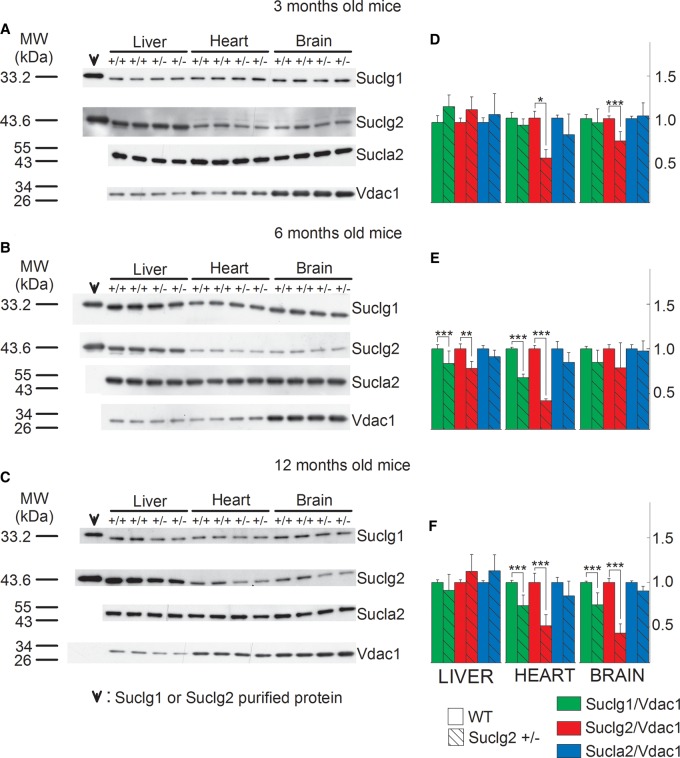
SUCL subunit expression in WT vs. Suclg2+/− mice. (**A**–**C**) Scanned images of Western blotting of purified SUCLG1 and SUCLG2 and mitochondria of 3-, 6- and 12-month-old WT and Suclg2+/− mice from liver, heart and brain. (**D**–**F**) Band density quantification of the scanned images shown in **A**–**C**, respectively. Data were arbitrarily normalized to the average density of the first two bands of WT mice per organ. *P < 0.05, **P < 0.01 and ***P < 0.001. Each Western blot lane contains mitochondria (except those containing the purified SUCLG1 or SUCLG2 proteins) pooled from two or four organs per animal group. Data shown in the bar graphs are SEM.

Mitochondria were prepared from the livers, hearts and brains of 3-, 6- and 12-month-old WT and Suclg2+/− mice, and SUCLG1, SUCLA2, SUCLG2 and VDAC1 were immunodetected by Western blotting, exactly as described in Figure 2. Scanned images of representative Western blots are shown in Figure 5A–C. As shown in the first lane of the left topmost panel in Figure 5A, purified recombinant SUCLG1 or SUCLG2 protein has been immunodetected. Purified protein has been loaded in the leftmost lane (30 ng). From the bands obtained from the purified proteins in relation to those obtained from the purified mitochondria, we deduce that (i) the bands detected from the mitochondrial samples corresponding to slightly lower though nearly identical MW presumably due to the hexahistidine tags of the recombinant proteins genuinely represent the sought proteins and (ii) the amount of either SUCLG1 or SUCLG2 in 3.75 µg of purified mitochondria corresponds to slightly <30 ng. Anti-VDAC1 was used as a loading control.

As shown in Figure 5A–C and from the quantification of the band densities in relation to that of VDAC1 shown in Figure 5D–F, respectively, Suclg2+/− mice exhibited up to 56% decrease in Suclg2 expression, a mostly insignificant decrease in Suclg1 expression, and no rebound increase in Sucla2 expression. The variability in the decrease in Suclg2 (and Sucla2) expression in these transgenic mouse lines probably reflects the ‘leakiness’ of the mutant allele that could produce WT mRNAs by alternative splicing around the gene trap cassette, as has been shown in several similar situations [22,56,61,75].

From the above experiments, we obtained the information that deletion of one *Suclg2* allele was not associated with the rebound effects as seen in the Sucla2+/− mice. Because of the lack of a rebound effect on Sucla2 expression in Suclg2+/− mice, in conjunction with the fact that SUCLG2 deficiency has never been reported in humans this transgenic strain was further investigated only regarding respiration and SLP during chemical or true anoxia.

### Characterization of SUCL subunit expression and enzymatic activities of Sucla2+/−/Suclg2+/− double heterozygote mice

**Figure 6.**
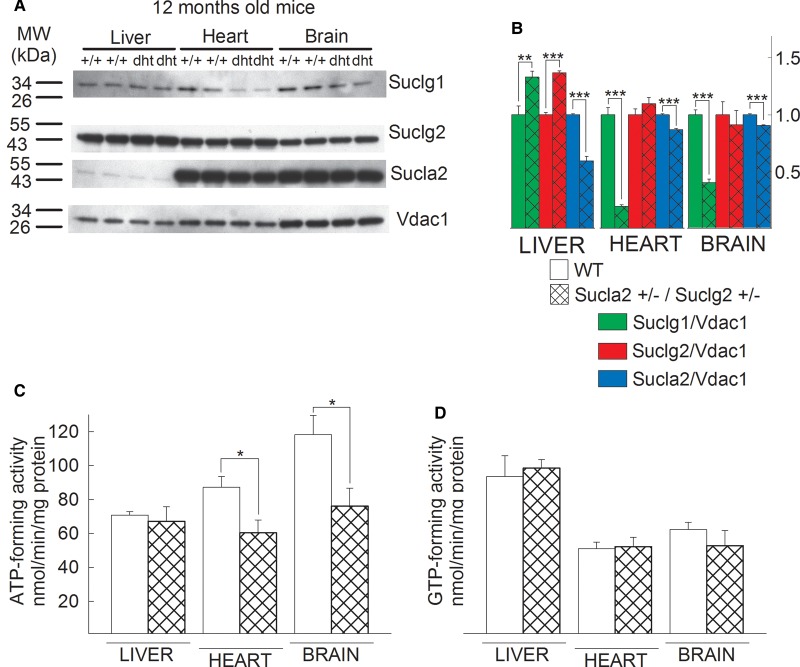
Characterization of SUCL subunit expression of Sucla2+/−/Suclg2+/− mice (dht: double heterozygote) and SUCL enzymatic activities. (**A**) Scanned images of Western blotting of 12-month-old WT and Sucla2+/− / Suclg2+/− mice from liver, heart and brain. (**B**) Band density quantification of the scanned images shown in **A**. Data were arbitrarily normalized to the average density of the first two bands of WT mice per organ. *P < 0.05, **P < 0.01 and ***P < 0.001. Each Western blot lane contains mitochondria pooled from two or four organs per animal group. Data shown in the bar graph are SEM. (**C**) Bar graphs of ATP-forming SUCL activity from mitochondria of 12-month-old WT (solid) and Sucla2+/−/Suclg2+/− (double-striped) mice from liver. (**D**) Bar graphs of GTP-forming SUCL activity from mitochondria of 12-month-old WT (solid) and Sucla2+/−/Suclg2+/− (double-striped) mice from liver. Data shown in **C** and **D** are SEM from two or four pooled organs per group from four independent experiments.

Since the deletion of one *Sucla2* allele led to a rebound increase in Suclg2 expression, we investigated the effect of combined loss of one allele from each *Sucla2* and *Suclg2* gene. Thus, we cross-bred Sucla2+/− mice with Suclg2+/− mice, which yielded viable Sucla2+/−/Suclg2+/− offspring. The results of Western blotting of mitochondria isolated from the brains, livers and hearts of 12-month-old WT vs. Sucla2+/−/Suclg2+/− mice probing for SUCLG1, SUCLG2 and SUCLA2 (and VDAC1 as a loading control) are shown in Figure 6A (performed exactly as described in Figure 2). The quantification of the band densities in relation to that of VDAC1 is shown in Figure 6B. As shown in Figure 6B, deletion of one *Sucla2* allele still yields a rebound increase in Sucgl2 expression in liver, albeit protracted because these mice also lack one *Suclg2* allele. By the same token, the anticipated decrease (due to deletion of one *Suclg2* allele) in Suclg2 expression is lost, presumably because of the effect(s) of deletion of the *Sucla2* allele, antagonizing the diminution in expression of Suclg2. These results are also reflected in the measured ATP- and GTP-forming activities of WT vs. Sucla2+/−/Suclg2+/− mice, shown in Figure 6C,D, respectively: ATP-forming activity is diminished in the double heterozygote mice, compared with WT littermates due to loss of one *Sucla2* allele; however, GTP-forming activity remains unaffected, despite the loss of one *Suclg2* allele.

From these experiments, we deduce that the effect(s) of deleting one *Sucla2* allele up-regulating Suclg2 expression is so dominant that it adequately antagonizes or even supersedes the effect(s) of a concomitant loss of one *Suclg2* allele.

### The effect of deleting one Sucla2 allele on mitochondrial respiration

**Figure 7.**
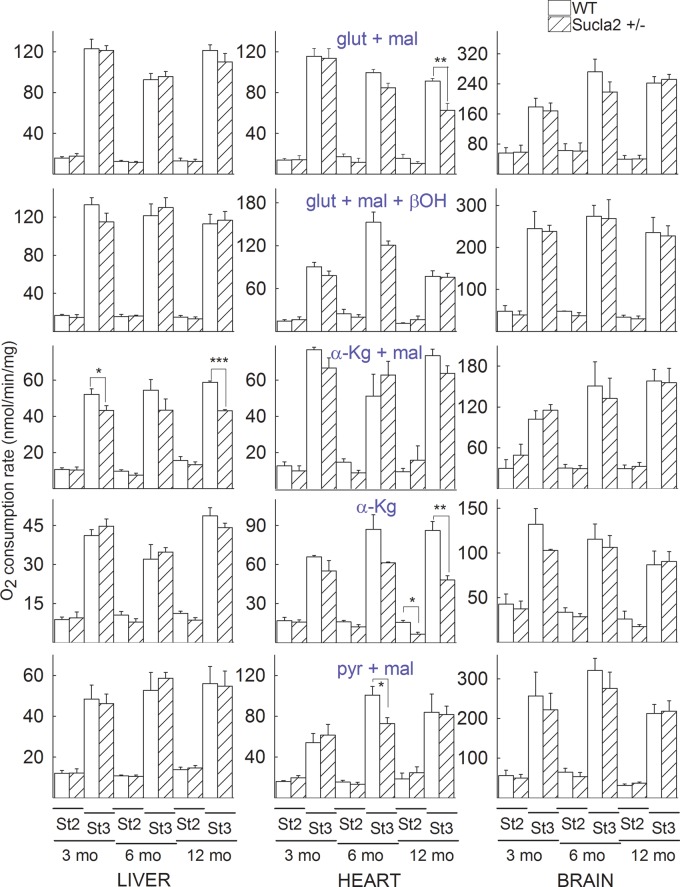
Bar graphs of measurements of oxygen consumption in the medium containing isolated mitochondria of 3-, 6- and 12-month-old WT (solid) and Sucla2+/− (striped) mice from liver, heart and brain. Substrate combinations used as indicated in the panels, all at concentration of 5 mM, except β-hydroxybutyrate (β-OH) that was 4 mM. St2: state 2 respiration; St3: state 3 respiration. *P < 0.05, **P < 0.01 and ***P < 0.001. Data shown are SEM from two or four pooled organs per animal group from four independent experiments.

Mitochondria were prepared from the livers, hearts and brains of 3-, 6- and 12-month-old WT and Sucla2+/− mice and states 2 and 3 (induced by the addition of 2 mM ADP) of mitochondrial respiration were evaluated using an array of substrates, as indicated in Figure 7. As shown in Figure 7, with the exception of one combination for state 2 respiration and five combinations for state 3 respiration, the remaining 84 combinations of substrates per tissue of origin per age of mice did not reveal statistically significant differences between WT and Sucla2+/− mice.

From these experiments, we concluded that a partial decrease in Sucla2 expression did not impact negatively on mitochondrial respiration to an appreciable extent. This lack of effect could be also explained by the rebound increases in Suclg2 expression and associated increases in GTP-forming SUCL activity that in turn could impact on mitochondrial ATP output through the concerted action of the nucleotide diphosphate kinase. In addition to this, it is possible that the flux control coefficient of SUCL regarding mitochondrial respiration is small enough, so that inhibition of this enzyme to the extent observed hereby in the Sucla2+/− transgenic mice was insufficient to warrant a measurable effect on mitochondrial respiration.

### The effect of deleting one Sucla2 allele on ΔΨm and SLP during inhibition of complex I by rotenone or true anoxia

In the absence of oxygen or when the electron transport chain is impaired, SLP in the matrix substantiated by the reaction catalyzed by SUCL is the only means for production of high-energy phosphates in mitochondria. When the electron transport chain is compromised and F_0_–F_1_ ATP synthase reverses pumping protons out of the matrix at the expense of ATP hydrolysis, the mitochondrial membrane potential is maintained — albeit at decreased levels — for as long as matrix SLP is operational, without a concomitant reversal of the adenine nucleotide translocase (ANT), thus preventing mitochondria from becoming ATP consumers [9,10,12,13]. Mitochondrial SLP can be assessed by recording the directionality of the ANT during respiratory inhibition. The latter can be achieved either pharmacologically (i.e. by inhibiting complex I with rotenone) or with anoxia. The assessment of the directionality of the ANT can be performed by a ‘biosensor test’ developed in our laboratory [13]. This test is based on the concept that one molecule of ATP^4−^ is exchanged for one molecule of ADP^3−^ (both nucleotides being Mg^2+^-free and deprotonated) by the ANT [37]. Therefore, during forward mode of the ANT (ATP moving outward from mitochondria in exchange of an inward movement of one ADP), abolition of its operation by a specific inhibitor such as cATR leads to an increase in ΔΨm, whereas during the reverse mode of ANT, cATR leads to a loss of ΔΨm.

In this work, we evaluated matrix SLP during either inhibition of complex I by rotenone or during anoxia. Mitochondria were prepared from the livers, hearts and brains of 3-, 6-, and 12-month-old WT (black traces) and Sucla2+/− (red traces) mice, and ΔΨm was evaluated using an array of substrates, indicated in Supplementary Material.

**Figure 8.**
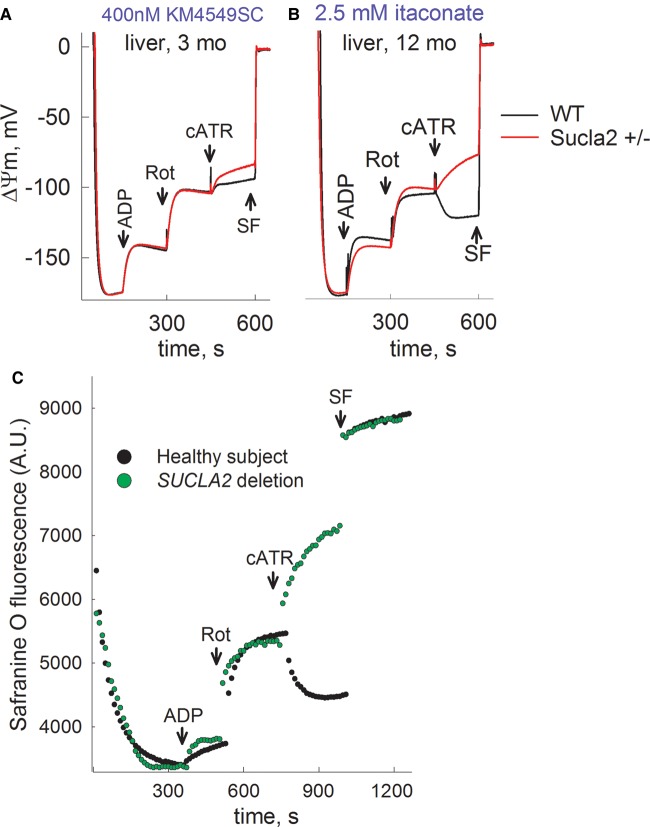
Reconstructed time courses of safranin O signal calibrated to ΔΨm (solid traces), and parallel measurements of oxygen concentration in the medium (dotted traces) in mitochondria of 3- and 12-month-old WT (black) and Sucla2+/− (red) mice isolated from liver, in the presence of (A) 400 nM KM4549SC or (B) itaconate (2.5 mM). ADP: 2 mM; cATR, 1 µM. Substrate concentrations were glutamate (5 mM) and malate (5 mM). At the end of each experiment, 1 µM SF 6847 was added to achieve complete depolarization. (**C**) Reconstructed time course of safranin O signal from permeabilized fibroblasts of a control subject (black dots) and a patient suffering from complete SUCLA2 deletion (green dots). Rot: rotenone, 1 µM. Data shown are representative of at least four independent experiments.

Time-lapse recordings of safranin O fluorescence reflecting ΔΨm (while measuring oxygen concentration in the same sample) were achieved by using the recently developed O2k-Fluorescence LED2-Module of the Oxygraph-2k (Oroboros Instruments, Innsbruck, Austria). Mitochondria were allowed to deplete the oxygen dissolved in the air-sealed chamber and additions of chemicals through a tiny-bore hole did not allow re-oxygenation of the buffer from the ambient atmosphere. The sequences of additions were as follows: mitochondria were added in 2 ml of buffer (see ‘Experimental Procedures’) containing substrates as indicated in the panels and allowed to fully polarize (solid traces). State 3 respiration was initiated by ADP (2 mM) depolarizing mitochondria; within a few minutes (depending on the substrates), mitochondria became anoxic as verified by recording ‘zero’ levels of dissolved oxygen in the chamber (dotted traces). Anoxia also coincided with the onset of an additional depolarization leading to a clamp of ΔΨm. The subsequent addition of cATR (1 µM) caused either a moderate re- or de-polarization, implying that the ANT was operating in the forward or reverse mode, respectively. Further addition of the uncoupler SF 6847 (1 µM) was subsequently used to cause a complete collapse of ΔΨm and assist in the calibration of the fluorescence signal. As shown in Supplementary Figures S1A–C and S2C,D, there were no differences between mitochondria from WT and Sucla2+/− mice. Likewise, when SLP was examined during inhibition of the respiratory chain by rotenone instead of anoxia, no differences between WT and Sucla2+/− mice mitochondria were observed (Supplementary Figures S2A,B and S3A–C). Similar to the results obtained from mitochondrial respiration, the lack of effect could be also explained by the rebound increases in Suclg2 expression and associated increases in GTP-forming SUCL activity that in turn could affect mitochondrial ATP output through the concerted action of the nucleotide diphosphate kinase (see above), or, as mentioned above, it is possible that the flux control coefficient of SUCL regarding mitochondrial respiration is small enough, so that inhibition of this enzyme to the extent observed hereby in the Sucla2+/− transgenic mice was insufficient to warrant a measurable effect. By the same token, no differences in mitochondrial respiration or SLP during chemical or true anoxia were observed by comparing Suclg2+/− vs. WT littermate mice (Supplementary Figures S4–S6). By comparing WT vs. Sucla2+/−/Suclg2+/− double heterozygote mice, we also observed no difference in the ability of SLP to maintain ANT in the forward mode, with the exception of using glutamate + malate + β-hydroxybutyrate as substrates (a substrate combination that does not favor SLP) in liver mitochondria, where we obtained the full spectrum of results, ranging from maintenance of SLP to its abolition (Supplementary Figure S7). However, a concomitant submaximal inhibition of SUCL by KM4549SC or itaconate [51] revealed that mitochondria obtained from Sucla2+/− mice are less able to perform SLP (Figure 8A,B and Supplementary Figures S8–S10). The argument that SUCLA2 is critical for SLP is also strengthened by the findings where, by applying the ‘biosensor test’ in permeabilized fibroblasts from a control subject vs. a patient suffering from complete deletion of *SUCLA2* [47], the *in situ* mitochondria from the patient are unable to perform SLP during respiratory inhibition by rotenone (Figure 8C).

### The effect of deleting one Sucla2 allele on ETC/CS

**Figure 9.**
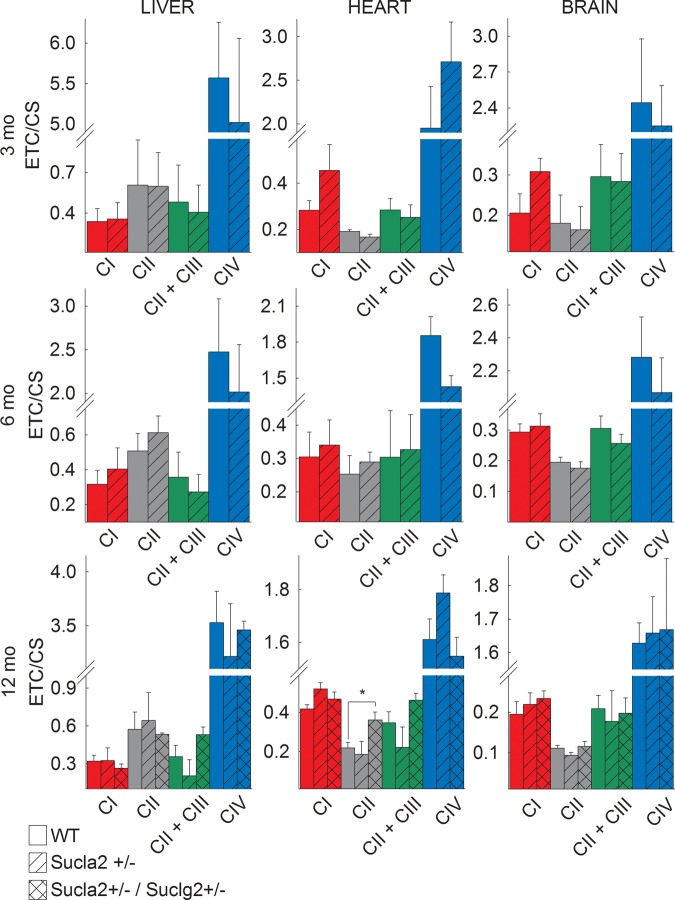
Bar graphs of measurements of complex I, II, II + III and IV activities (ETC) ratioed to CS activity in isolated mitochondria of 3-, 6- and 12-month-old WT (solid), Sucla2+/− (striped) mice from liver, heart and brain, and Sucla2+/−/Suclg2+/− (double-striped) mice from livers of 12-month-old mice. *P < 0.05. Data shown are SEM from two or four pooled organs per animal group from four independent experiments.

Mindful that some patients suffering from SUCLA2 deficiency exhibited decreases in the activities of electron transport chain complexes, we investigated the effect of deleting one *Sucla2* allele in mice on complex I, II, II/III and IV activities, ratioed to citrate synthase activity. As shown in Figure 9, mitochondria from all tissues and all ages revealed no statistically significant differences between WT and Sucla2+/ mice. However, by comparing WT vs. Sucla2+/−/Suclg2+/− double heterozygote mice, there was a statistically significant increase in succinate dehydrogenase activity in heart mitochondria, echoing the results of Donti et al. [18] in Sucla2−/− mouse embryonic fibroblasts.

### The effect of deleting one Sucla2 allele on mtDNA

**Figure 10.**
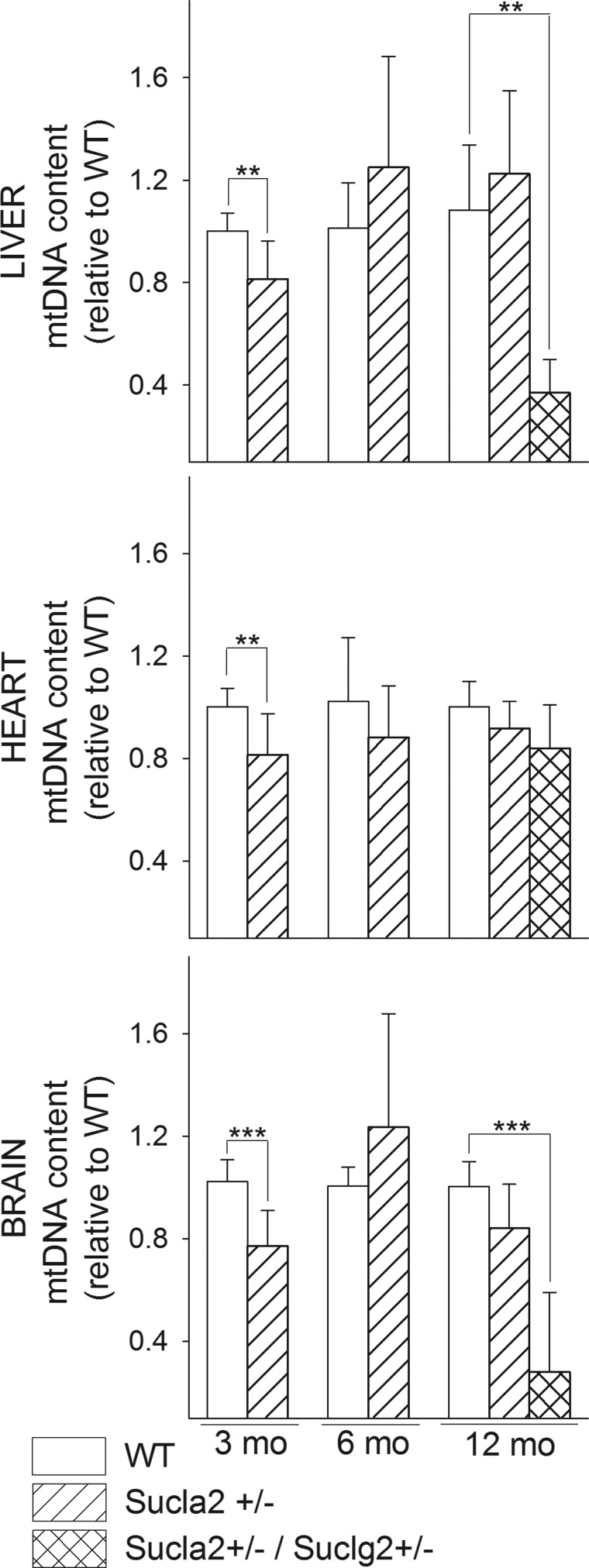
Bar graphs of relative measurements of mtDNA content of livers, hearts and brains from 3-, 6- and 12-month-old WT (solid) compared with that from Sucla2+/− (striped) mice, and Sucla2+/−/Suclg2+/− (double-striped) mice from livers of 12-month-old mice. **P < 0.01 and ***P < 0.001. Data shown are SD from four pooled organs per animal group from four independent experiments.

Because of the involvement of SUCL in the maintenance of mtDNA, we compared the amount of mtDNA in the tissues of WT vs. Sucla2+/− mice. As shown in Figure 10, relative mtDNA content from the livers, hearts and brains of 3-, 6- and 12-month-old mice was quantitated by real-time PCR. It is evident that there is a moderate but statistically significant decrease in mtDNA in all tissues of 3-month-old mice and in the brains of 12-month-old mice. Furthermore, by comparing WT vs. Sucla2+/−/Suclg2+/− double heterozygote mice, there was a much greater statistically significant decrease in mtDNA in the livers and brains of double heterozygote mice, compared with WT littermates.

### The effect of deleting one Sucla2 allele on blood carnitine esters

**Figure 11.**
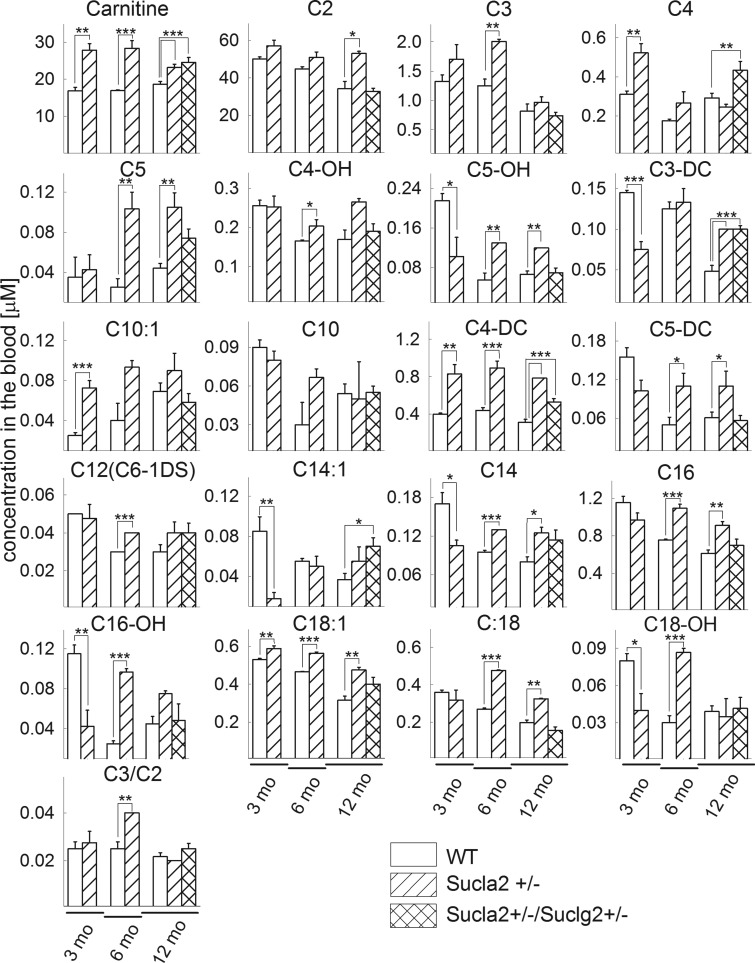
Bar graphs of measurements of carnitine and its esters in the blood of 3-, 6- and 12-month-old WT (solid) and Sucla2+/− (striped) mice and Sucla2+/−/Suclg2+/− (double-striped) from 12-month-old mice. *P < 0.05, **P < 0.01 and ***P < 0.001. Data shown are SEM from four blood draws per animal group from four mice each. Carnitine (free); C2(acetyl); C3 (propionyl); C4 (butyryl/isobutyryl); C5 (isovaleryl/2-methylbutyryl/pivaloyl); C4-OH (3-hydroxybutyryl); C5-OH (3-hydroxy isovaleryl/2-methyl 3-hydroxybutyryl); C3-DC (malonyl); C10:1 (decenoyl); C10 (decanoyl); C4-DC (methylmalonyl/succinyl); C5-DC (glutaryl); C12 (C6-1DS, dodecanoyl); C14:1 (tetradecenoyl); C14 (myristoyl); C16 (palmitoyl; C16-OH (3-hydroxyhexadecenoyl); C18:1 (oleyl); C18 (stearoyl); C18-OH (3-hydroxystearoyl).

Finally, in view of the association of SUCL activity with the catabolism of a particular group of biomolecules converging to succinyl-CoA through propionyl-CoA and methylmalonyl-CoA which are in equilibrium with their carnitine esters, we measured the levels of 20 carnitine esters in the blood of mice. As shown in Figure 11, there are statistically significant increases in 36 out of 63 comparisons of carnitine esters in the blood of Sucla2+/− mice from all age groups compared with that from WT mice, but also 6 occasions in which carnitine esters of Sucla2+/− mice is decreased compared with those of WT mice. What is also noteworthy is that although SUCLA2 deficiency in humans is associated with elevations of C3 and C4-DC levels, in the Sucla2+/− mice there was elevation of several additional esters including those encompassing long-chain fatty acid chains (C16-OH, C18:1, C:18 and C18-OH). This may be due to a plausible ‘CoASH trap’ in the form of succinyl-CoA, depleting mitochondria from CoASH which is critical for the entry and catabolism of long-chain fatty acids in mitochondria. Indeed, as seen in oxygen consumption experiments (Figure 7) when using α-ketoglutarate (α-Kg) as a substrate, heart mitochondria (which are dependent on CoASH for optimal catabolism of fatty acids) of Sucla2+/− mice exhibited smaller state 2 and state 3 respiration rates than WT mice, implying that KGDHC activity may be impaired, possibly due to insufficient amounts of CoASH. Furthermore, by comparing WT vs. Sucla2+/−/Suclg2+/− double heterozygote mice, there was a greater statistically significant increase in some carnitine esters of the blood of double heterozygote mice, compared with WT littermates.

## Discussion

Mitochondrial diseases are collectively considered as a major cause of encephalomyopathies and other multisystem maladies [19,62,63]. A considerable fraction of this pool of diseases encompasses mtDNA depletion [23]. Many animal models have been generated to model mtDNA depletion by specifically deleting genes important for mtDNA replication [2,25,27,34,43,45,73,74], although only one study has addressed the role of SUCL [18]. In the latter work by Donti et al., tissues from Sucla2−/− mice were examined at an embryonic stage, because —like in our study— no viable homozygote offsprings were born. Nevertheless, accordance to a large extent is observed between the results obtained by Donti et al. and our study, despite embryonic tissues of Sucla2−/− mice being examined as opposed to here where we investigated tissues from Sucla2+/− heterozygote adult mice, those engineered to lack one Suclg2 allele, as well as Sucla2+/−/Suclg2+/− double heterozygotes. Among the striking similarities are the varying degrees of mtDNA alterations, the reciprocal increase in Suclg2 expression when Sucla2 levels are decreased, elevations in methylmalonyl esters and a mild increase in succinate dehydrogenase activity. However, unlike in Donti et al. [18], we did not observe alterations in the activities of other than complex II ETC components, or any changes in mitochondrial respiration, or SLP.

In more detail, the most important results of the present work are those obtained by the comparisons of WT with Sucla2+/− mice. These can be categorized to: (i) rebound increase in Suclg2 expression and associated GTP-forming activity; (ii) lack of effect on bioenergetic parameters including SLP; (iii) moderate mtDNA decrease and (iv) elevation of short- and long-chain carnitine esters in the blood of heterozygote mice. It is important to emphasize that the rebound increase in Suclg2 expression in Sucla2+/− mice seemed to occur mostly in heart mitochondria, also in brain mitochondria but only from the older (6–12 months old) mice and not in liver mitochondria. Furthermore, the increase in GTP-forming activity also seemed heart-specific, as well as the changes in complex II activity observed in Sucla2+/−/Suclg2+/− mice. Obviously, the molecular mechanisms responsible for these rebound effects are tissue-specific and appear to be operational in the heart and at least some brain-specific cells, but not in the liver. The elucidation of such molecular mechanisms may be of great value in setting an example of gene–gene interactions of similar nature.

On the same line, regarding the rebound increase in Suclg2 expression and associated GTP-forming activity in Sucla2 heterozygote mice, the concept of ‘complementation’ between Sucla2 and Suclg2 and —as an extension of this— ATP- and GTP-forming activity has been proposed earlier [47]. As mentioned above, this has been observed in embryonic tissues of Sucla2−/− mice [18], but not in fibroblasts from patients exhibiting mutations in *SUCLA2* [47]. However, as is evident from our results, this complementation is sufficient to alleviate only some of the biochemical abnormalities of the Sucla2 heterozygous mice. Indeed, mitochondrial respiration, ΔΨm, ETC/CS activities and SLP were virtually identical between WT and Sucla2+/− mice, while there was mild mtDNA depletion but significant alterations in blood carnitine esters. The alterations in mtDNA should be attributed to alterations in the activity of SUCL with caution; it has been recently reported that GABA transaminase is essential for mitochondrial nucleoside metabolism and thus is important for mtDNA maintenance, and it co-immunoprecipitates with SUCLG1, SUCLG2 and SUCLA2 [5]. The alterations in the carnitine esters concern those with both short- and long-chain. However, the levels of free carnitine in four heterozygous human carriers for *SUCLA2* mutations were not significantly elevated, compared with reference values (values were 25, 27, 29 and 37, with reference values of 24–64 mmol/l).

Finally, regarding the rebound increase in Suclg2 expression in Sucla2+/− mice, it is noteworthy to emphasize that the concomitant loss of one *Suclg2* allele yielding Sucla2+/−/Suclg2+/− double transgenic mice was not sufficient to impose a considerable decrease in Suclg2 expression or GTP-forming SUCL activity. Apparently, the translational or posttranslational mechanism(s) mediating this effect (as there was no change in *Suclg2* transcript, reported in ref. [18]) supersede those occurring at the gene level and merit further consideration.

In aggregate, we profiled the metabolism of two transgenic mouse models for β-subunit components of SUCL; the results presented and the availability of these transgenic mouse colonies to the scientific community are of value in the pursuit for understanding SUCL deficiency.

## Abbreviations

β-OH, beta-hydroxybutyrate; ANT, adenine nucleotide translocase; cATR, carboxyatractyloside; CS, citrate synthase; DTNB, 5,5′-dithiobis(2-nitrobenzoic acid); ETC, electron transport chain; SLP, substrate-level phosphorylation; SUCL, succinate-CoA ligase; VDAC1, voltage-dependent anion channel 1; ΔΨm, mitochondrial membrane potential.

## Author Contribution

G.K., D.R., J.D., B.N., O.M., A.S., P.I., C.M., E.O., I.I., D.A., Z.V., M.A., K.A., M.N., H.I., A.G., M.J.M, Z.N. and C.C. performed the experiments. C.C. designed the experiments, evaluated the data and wrote the manuscript. A.P. and V.A.-V. edited the manuscript.

## Funding

This work was supported by the Lendület grant LP2012-39/2012 of the Hungarian Academy of Sciences to László Csanády, the Danish National Health Research Council grant [12-127702 to E.O.]; a compensatory Hadassah research grant to A.S. [TÁMOP 4.2.1./B-09/1/KMR and BIOINF09TÉT_10-1-2011-0058 to M.J.M.]; the Országos Tudományos Kutatási Alapprogram (OTKA) grant [81983], the Hungarian Academy of Sciences grant [02001], the Hungarian Brain Research Program [KTIA_13_NAP-A-III/6] to V.A.-V., a grant of MTA-SE ‘Hereditary’ Endocrine Tumours Research Group to A.P. and grants MTA-SE Lendület Neurobiochemistry Research Division [95003], OTKA [NNF 78905], OTKA [NNF2 85658] and OTKA [K 100918] to C.C.
